# Influence of verapamil on the pharmacokinetics of rotundic acid in rats and its potential mechanism

**DOI:** 10.1080/13880209.2021.1871634

**Published:** 2021-02-17

**Authors:** Haihua Shang, Ze Wang, Hong Ma, Yinghui Sun, Xiaoyan Ci, Yuan Gu, Changxiao Liu, Duanyun Si

**Affiliations:** a School of Pharmacy, Shenyang Pharmaceutical University, Shenyang, China; b State Key Laboratory of Drug Delivery and Pharmacokinetics, Tianjin Institute of Pharmaceutical Research, Tianjin, China; c College of Traditional Chinese Medicine, Tianjin University of Traditional Chinese Medicine, Tianjin, China; d Research Unit for Drug Metabolism, Chinese Academy of Medical Sciences, Tianjin, China

**Keywords:** Drug–drug interaction, CYP3A, P-gp, oral bioavailability

## Abstract

**Context:**

Rotundic acid (RA), a plant-derived pentacyclic triterpene acid, has been reported to possess extensive pharmacological activities. The poor bioavailability limits its further development and potential clinic application.

**Objective:**

To clarify the potential mechanism for poor oral bioavailability.

**Materials and methods:**

The single-dose pharmacokinetics of orally administered RA (10 mg/kg) in Sprague–Dawley rats without or with verapamil (25 or 50 mg/kg) were investigated. Additionally, MDCKII-MDR1 and Caco-2 cell monolayers, five recombinant human cytochrome P450 (rhCYP) enzymes (1A2, 2C8, 2C9, 2D6 and 3A4), and rat liver microsomes were also conducted to investigate its potential mechanism.

**Results:**

Verapamil could significantly affect the plasma concentration of RA. Co-administered verapamil at 25 and 50 mg/kg, the AUC_0–∞_ increased from 432 ± 64.2 to 539 ± 53.6 and 836 ± 116 ng × h/mL, respectively, and the oral clearance decreased from 23.6 ± 3.50 to 18.7 ± 1.85 and 12.2 ± 1.85 L/h/kg, respectively. The MDCKII-MDR1 cell assay showed that RA might be a P-gp substrate. The rhCYPs experiments indicated that RA was mainly metabolized by CYP3A4. Additionally, verapamil could increase the absorption of RA by inhibiting the activity of P-gp, and slow down the intrinsic clearance of RA from 48.5 ± 3.18 to 12.0 ± 1.06 µL/min/mg protein.

**Discussion and conclusions:**

These findings indicated that verapamil could significantly affect the pharmacokinetic profiles of RA in rats. It was demonstrated that P-gp and CYP3A were involved in the transport and metabolism of RA, which might contribute to the low oral bioavailability of RA.

## Introduction

Rotundic acid (RA) is an ursane-type of pentacyclic triterpene acid isolated from the dried barks of *Ilex rotunda* Thunb. (Aquifoliaceae), which is officially named as Ilicis Rotundae Cortex (IRC) and is widely used in clinical therapy and daily health in China (Chinese Pharmacopoeia Commission 2015; Liu and Yin 2016; Yang et al. 2020). Triterpenes are the major bioactive compounds obtained from IRC along with saponins, flavonoid glycosides, phenolic acids, and steroids (Yang, Li, Ruan, Tong, et al. 2018). RA is a representative bioactive pentacyclic triterpene in IRC that has been selected as the quality control marker (Zhu et al. 2015; Yang, Li, Ruan, Xue, et al. 2018). RA possesses numerous and comprehensive pharmacological activities, including anticancer, anti-inflammatory, antimycobacterial, antibacterial, antidiabetic, lipid-lowering properties, etc. (Hsu et al. 2015; Nguyen et al. 2017; Aro et al. 2019; Han et al. 2019; Roy et al. 2019; Yan et al. 2019). In addition, RA combined with radiation therapy can increase the efficacy of radiotherapy and reduce the side-effects via the ATM/p53 pathway (Wang et al. 2018).

Pentacyclic triterpene acids (PTAs) are generally considered to have low bioavailability due to the poor solubility and dissolution rate, low intestinal absorption, potential efflux transport, first-pass metabolism, etc. It has previously been reported (Lee et al. 2011; Zhang et al. 2018; Liu et al. 2019) that efflux pump P-glycoprotein (P-gp) and cytochrome P450 (CYP450) enzymes CYP3A located in the intestine and liver have the potential to affect the disposition of TCM and drug–drug interaction (DDI) *in vivo*. Therefore, in addition to their own physicochemical properties and pharmaceutical dosage form, CYP450 enzymes and efflux transporters play a major role in the assessment of bioavailability. For example, the bioavailability of glycyrrhizin in rats is 4.0% due to the poor mucosal permeability and first-pass elimination (Wang et al. 1994). With poor absorption, extensive metabolic clearance, and involvement of P-gp efflux transport, the absolute oral bioavailability of oleanolic acid in rats was 0.7% (Jeong et al. 2007; Yang et al. 2017). It was reported in a previous study that the oral bioavailability of RA was only 4.52% after oral administration in rats (Nan et al. 2016). However, to date, there is no information available regarding the metabolic stability and intestinal absorption characteristics of RA, and the reason for its poor bioavailability. We hypothesized the low oral bioavailability of RA is due to P-gp mediated drug efflux and/or CYP3A mediated metabolism of RA as the same as PTAs.

Verapamil (Ver), a calcium channel blocker to treat hypertension, has been reported as an inhibitor of both CYP3A and P-gp and used for DDI studies *in vivo*. While it has been proved to relate to the metabolism and absorption of the victim drugs (Srinivas 2008; Wang et al. 2016). With the extensive pharmacological activity and significant clinical efficacy, IRC has been used in daily health care for a long time, no research reports were also currently available related to DDI between RA and other drugs. Whether verapamil can affect the *in vivo* pharmacokinetics of RA is still unknown, and the potential absorption mechanism of RA, when co-administered with verapamil *in vitro*, remains to be elucidated.

Thus, the effects and potential mechanisms of verapamil on the pharmacokinetics of RA were investigated, which could clarify the main reasons for its poor oral bioavailability, and provide information related to the further development and potential clinical application of RA. After pre-treatment with or without verapamil, the *in vivo* pharmacokinetics of RA in rats was determined using a rapid, simple, and reliable LC-MS/MS method. Additionally, using empty vector-transfected Madin-Darby canine kidney type II (MDCKII-Mock) epithelial cells, MDCKII cells with overexpressed human MDR1 gene encoding P-gp (MDCKII-MDR1), the human colonic Caco-2 cell lines, five recombinant human cytochrome P450 (rhCYP) enzymes (1A2, 2C8, 2C9, 2D6 and 3A4), and rat liver microsomes (RLMs), the potential absorptive and metabolic mechanisms of RA were investigated.

## Materials and methods

### Chemicals and reagents

Verapamil hydrochloride (purity greater than 99.0%), β-nicotinamide adenine dinucleotide phosphate (NADPH), Lucifer yellow, and rhodamine-123 were obtained from Sigma-Aldrich (St. Louis, MO, USA). Rotundic acid (purity greater than 98.6%) was purchased from Chengdu Pufei De Biotech Co., Ltd. (Sichuan, China). Etofesalamide (Internal Standard, IS) was obtained from the National Institutes for Food and Drug Control (Beijing, China). Rat liver microsomes, rhCYP 1A2, 2C8, 2C9, 2D6 and 3A4 were provided from the Research Institute for Liver Disease (Shanghai) Co., Ltd. (Shanghai, China). Dulbecco’s Modified Eagle Medium (DMEM), penicillin (10,000 U/mL)-streptomycin (10 mg/mL), non-essential amino acids (NEAA), and foetal bovine serum (FBS) were acquired from GIBCO (Grand Island, NY, USA). CellTiter 96 Aqueous One Solution Cell Proliferation Assay (MTS) kit was provided from Promega Corp. (Madison, WI, USA). The HPLC grade acetonitrile and methanol were purchased from Thermo Fisher Scientific (China) Co., Ltd. (Shanghai, China). The analytical grade ammonium formate used in this experiment was procured from Tianjin Guangfu Fine Chemical Research Institute (Tianjin, China). Highly pure deionized water was prepared in-house using the BM-40 water purification system from Zhongsheng Maoyuan Tech. Co., Ltd. (Beijing, China). All other chemicals and reagents were of analytical grade and commercially available.

### Animal experiments

Male Sprague–Dawley rats (180–220 g) were purchased from SiPeiFu (Beijing) Biotechnology Co., Ltd. (Beijing, China). Rats were housed in a breeding room at stable temperature (22 ± 3 °C), humidity (55 ± 10%), and under non-pathogenic conditions. All animals were placed under the above conditions and acclimatized to the facilities for one week, then fasted for 12 h with water *ad libitum* before the experiments. The protocols involving experimental animals were carried out by the guidelines of the Animal Ethical Committee of Tianjin Institute of Pharmaceutical Research (Tianjin, China).

### *In vivo* pharmacokinetic study

To evaluate the influence of verapamil on the *in vivo* pharmacokinetics of RA, the male Sprague–Dawley rats were randomly divided into three groups (*n* = 6), including a control group and two pre-treatment groups. The pre-treatment groups received verapamil orally by gavage at a dose of 25 and 50 mg/kg (dissolved in 5% gum arabicin at a concentration of 5 and 10 mg/mL, respectively) separately for 15 min before the administration of RA at a dose of 10 mg/kg (dissolved in 5% gum arabicin at a concentration of 2 mg/mL). The dose of verapamil selected was equated to the human maximum recommended daily dose of 240–480 mg for a 60 kg person following allometric conversion (US Food and Drug Administration 2005; Cao et al. 2007). The control group received equal amounts of the vehicle (5% gum arabicin) before the oral administration of RA. Whole blood samples (200 µL) were collected from the orbital venous plexus at 0.0833, 0.25, 0.5, 1, 1.5, 2, 3, 4, 6, 8, 10 and 24 h after oral administration. All samples were transferred into heparinized tubes and then centrifuged for 5 min at 12,000 rpm and 4 °C immediately. The supernatants of separated plasma samples were harvested and stored at −20 °C until LC-MS/MS analysis.

### Preparation of rat plasma samples

RA was extracted from the sample using a simple protein precipitation method with a 3-fold volume of an organic solvent consisting of methanol-acetonitrile (1:2, v/v). Briefly, a 50 µL aliquot of plasma sample was thawed at room temperature and was then precipitated by 50 µL of methanol and 100 µL of acetonitrile containing IS (10 ng/mL). The sample was mixed uniformly for 1 min, and then centrifuged for 10 min at 12,000 rpm and 4 °C. Subsequently, 10 µL supernatants were injected into the LC-MS/MS system for analysis.

### Determination of rotundic acid by LC-MS/MS

The separation and mass spectrometric analysis of the RA was achieved on a Waters ACQUITY^TM^ Ultra Performance LC system coupled with a Tandem Quadrupole (TQ) Detector equipped with an electrospray ionization (ESI) source. Chromatographic separation was performed at 40 °C with an ACQUITYUPLC BEH C8 column (1.7 µm, 2.1 × 50 mm, Waters Corp., USA). The mobile phase consisted of methanol-acetonitrile (50:50, v/v, mobile phase A), and 5 mM ammonium formate-methanol (90:10, v/v, mobile phase B). While the gradient elution procedure was employed for the separation of analytes at a flow rate of 0.3 mL/min. The optimized gradient elution program was as follows: 0 min 60% B, 3 min 10% B, 4 min 10% B, and 5 min 60% B. The injection volume was 10 µL at an autosampler temperature of 6 °C. The analytical run time for each sample was 5.0 min. The HPLC elution was injected into waste for the first 1.2 min, into the MS detector for the next 1 min, and back into waste for the remaining run time. RA and IS were monitored with the mass spectrometer by negative ESI source mode. The ionization source parameters were set as follows: 2.5 kV, 30 V, 120 °C, 350 °C, 50 L/h, and 650 L/h for capillary voltage, cone voltage, source temperature, desolvation temperature, cone gas, and desolvation gas, respectively. The optimal collision energy was operated at 30 V for RA and 20 V for IS. Argon was used as the collision gas. Samples were analyzed by multiple reaction monitoring (MRM) of the transitions of *m/z* 487.3 → 469.3 for RA and *m/z* 256.0 → 227.0 for IS, respectively. The dwell time was set at 0.2 s per transition. Data were acquired by MassLynx 4.1 software.

A lower limit of quantification (LLOQ) was found to be 10 ng/mL for RA, with acceptable precision and accuracy (< ±20%). A good linear of this detected method ranged from 10 to 2000 ng/mL for RA. Intra- and inter-day precisions were lower than 8.2% expressed as relative standard deviation (RSD), while accuracy was within ±6.5% in terms of relative error (RE) for analytes.

### Cell culture

MDCKII-Mock and MDCKII-MDR1 cells were given as a gift by Fuji Biomedix Co., Ltd. (Japan) and cultured in DMEM supplemented with 10% FBS and 1% antibiotic (10,000 U/mL penicillin and 10 mg/mL streptomycin). Caco-2 cell line, the human epithelial cell used in this assay, was purchased from the Cell Bank of the Chinese Academy of Sciences (Shanghai, China) and maintained between 30 and 45 passages. It was cultured according to the previous report (Guo et al. 2018; Xiang et al. 2020). Briefly, the Caco-2 cells were cultured in DMEM supplemented with 10% FBS, 1% NEAA, and 1% antibiotic (10,000 U/mL penicillin and 10 mg/mL streptomycin). All cells were maintained in a 5% CO_2_ atmosphere and 90% relative humidity at 37 °C.

For transport studies, the MDCKII-Mock, MDCKII-MDR1, and Caco-2 cells were seeded at a density of 2 × 10^5^ cells/mL on Costar Transwell inserts (1.12 cm^2^ surface, polycarbonate, 12 mm diameter, 0.4 µm pore size). MDCKII-Mock and MDCKII-MDR1 cells were cultured for 5–7 days in medium, for the first four days, the medium was replaced freshly every two days, and then daily. The Caco-2 cells were cultured for 19–21 days in the medium. For the first 2 weeks, the medium was replaced freshly every two days, and then daily. Before transport studies, the cytotoxicity of RA against MDCKII-Mock, MDCKII-MDR1, and Caco-2 cells was evaluated using MTS assays. The transepithelial electrical resistance (TEER) of the monolayer cells was measured by Millcell ERS-2 (Millipore, USA), and only the monolayer with TEER exceeding 150 Ω·m^2^ (MDCKII-Mock and MDCKII-MDR1 cells) and 400 Ω·m^2^ (Caco-2 cells) were used for the transport experiment. The membrane integrity and transport function of MDCKII-Mock, MDCKII-MDR1, and Caco-2 cell monolayers were further verified with Lucifer yellow and Rhodamine-123. The qualified monolayers were used for transport studies.

### Effects of verapamil on the transport of rotundic acid across MDCKII-Mock, MDCKII-MDR1, and Caco-2 cell monolayers

Before the transport studies, the media in both chambers of the transwell were replaced by warm (37 °C) Hank’s balanced salt solution (HBSS), and then the MDCKII-Mock, MDCKII-MDR1, and Caco-2 cell monolayers were incubated at 37 °C and 5% CO_2_ for 20 min. HBSS at pH 7.4 was used as the buffer for bidirectional transport of RA. Our transport studies included absorptive direction transport from the apical (AP) side to the basolateral (BL) side and secretory direction transport from the BL side to the AP side. In the absorptive direction transport studies, RA solution was loaded onto the AP side at a concentration of 25 µM by adding a volume of 0.5 mL, and then 1.5 mL of HBSS was added to the BL side. On the contrary, in the secretory direction transport studies, RA solution was loaded onto BL side at a concentration of 25 µM by adding volume 1.5 mL, and then 0.5 mL of HBSS was added to AP side. After administration, the transwell plate was placed in a thermostatic oscillator shaking at 60 rpm and 37 °C. At 10, 20, 40 and 60 min, respectively, 50 µL incubating sample aliquot was collected from the receiver chamber and immediately replaced by the same volume of fresh HBSS (37 °C). In addition, the membrane integrity of MDCKII-Mock, MDCKII-MDR1, and Caco-2 cell monolayers were evaluated with Lucifer yellow (30 µM) and TEER value, and the efflux activity of P-gp was validated using a typical P-gp substrate rhodamine-123 (10 µM). Adding 50 µM verapamil (Rautio et al. 2006) to both sides of the cell monolayers and pre-incubating for 20 min at 37 °C, the inhibitory characteristics of P-gp inhibitors on the RA transport in MDCKII-Mock, MDCKII-MDR1, and Caco-2 cells were investigated.

The parameters *P*_app_, *R*_E_, and N*R*_E_ were calculated using the following equation:

$$P_{\mathrm{app}}=(\Delta Q/\Delta t)\times [1/(A\times C_{0})]$$

$$R_{E}=P_{\mathrm{app}\;(\mathrm{BL}\to \mathrm{AP})}/P_{\mathrm{app}\;(\mathrm{AP}\to \mathrm{BL})}$$

$$NR_{E}=R_{E\;(\mathrm{MDCKII}-\mathrm{MDR}1)}/R_{E\;(\mathrm{MDCKII}-\mathrm{Mock})}$$

where, *P*_app_ is the apparent permeability coefficient (cm/s), Δ*Q*/Δ*t* is the linear appearance rate of RA on the receiver chamber (µmol/s), *A* is the membrane surface area (cm^2^), *C*_0_ is the initial concentration in the donor chamber (µM), *R*_E_ is efflux ratio, and N*R*_E_ is net efflux ratio. Each experiment was carried out in triplicate.

### The metabolism of RA through rhCYPs

The metabolic stability of RA was evaluated against five rhCYPs (1A2, 2C8, 2C9, 2D6 and 3A4). Incubation was taken place in 0.1 M sodium phosphate buffer (pH 7.4) containing 5 mM MgCl_2_, 1 mM NADPH, and 25 pM rhCYP enzyme protein at 37 °C in a total volume of 1 mL. After pre-incubated for 5 min, the reactions were started by the addition of RA methanol solution (final concentration of 2 µM). Aliquots of 50 µL incubation solution were collected from the reaction at 0, 5, 10, 20 and 30 min, and quenched with 50 µL ice-cold methanol (containing 0.5% formic acid) and 100 µL ice-cold acetonitrile containing IS (10 ng/mL). The heat-inactivated rhCYP3A4 were used as negative controls. All reactions were conducted in triplicate, followed by LC-MS/MS analysis. Results presented in percentage of drug remaining in incubation starting at 100% at time zero.

### Effects of verapamil on the metabolic stability of rotundic acid in rat liver microsomes

Low metabolic stability is a major barrier to further improve the oral bioavailability of the herb drugs. To gauge the medicinal chemistry value of RA, we evaluated their metabolic stability in RLMs with or without verapamil. The *in vitro* microsomal stability assay was conducted in triplicate in rat liver microsomal systems, which were supplemented with NADPH as a cofactor. The assay conditions and reaction mixtures were similar to the previous report with minor modifications (Li et al. 2016; Sun et al. 2019). Briefly, the incubation system (final volume of 1 mL) consisted of 1 mg/mL rat liver microsomal protein, 1 mM NADPH, and 5 mM MgCl_2_ in 0.1 M sodium phosphate buffer (pH 7.4). The incubation system was pre-incubated at 37 °C for 15 min and initiated by adding RA methanol solution (final concentration of 2 µM). The effects of verapamil on the metabolic stability of RA were investigated by adding 50 µM of verapamil (Ma et al. 2000; Lee and Kim 2013; Dinger et al. 2014) to RLMs and pre-incubating for 15 min at 37 °C. The final concentration of methanol (used for dissolving the substrate and inhibitor) was lower than 1% (v/v). Negative control was performed in parallel in a heat-inactivated liver microsomes incubation system to measure any chemical instability or non-enzymatic degradation. Aliquots of 50 µL incubation solution were collected from the reaction at 0, 5, 15, 30 and 60 min, and quenched with 50 µL ice-cold methanol (containing 0.5% formic acid) and 100 µL ice-cold acetonitrile containing IS (10 ng/mL). The samples were then vortexed for 1 min and centrifuged at 12,000 rpm for 10 min at 4 °C. The supernatants were collected and analyzed by LC-MS/MS.

Substrate depletion and *in vitro* half-life (*t*_1/2_) are common descriptors for metabolic stability. The *t*_1/2_ is calculated by the substrate disappearance. The obtained concentration value for time *t* is divided by the value for time zero and then multiplied by a factor of 100, which represents the ‘% Remaining’ to provide the relative remaining substrate level for the time *t*. The natural logarithm (ln) of the ‘% Remaining’ of RA was plotted against the incubation time (in min) using linear regression. The parameters *t*_1/2_ and intrinsic clearance (CL_int_) were obtained from the following equation:

$$t_{1/2}=0.693/k_{e}$$

CL_int_ (µL/min/mg protein) = 0.693/*t*_1/2_ × volume of incubation (µL)/protein in the incubation (mg).

Where, *k*_e_, the elimination rate constant, is the slope of a linear regression curve in a semilog plot. Data were collected from triplicate experiments.

### Data analysis

Pharmacokinetic parameters were calculated by Phoenix WinNonlin Version 7.0 (Certara, L.P.). The plasma concentration data were analyzed using the non-compartmental analysis to obtain primary pharmacokinetic parameters such as *t*_1/2_, AUC, *C*_max_, *T*_max_, the apparent oral clearance (CL/F), and the apparent volume of distribution (*V*_d_/*F*). All experimental results were presented as the mean ± standard deviation (SD). Statistical comparisons were analyzed by independent-samples *t*-test procedure and one-way analysis of variance (ANOVA) using IBM SPSS Statistics 25 software. It was considered to be statistically significant when the *p*-value was less than 0.05.

## Results

### Effect of verapamil on the pharmacokinetics of rotundic acid

**Figure 1.**
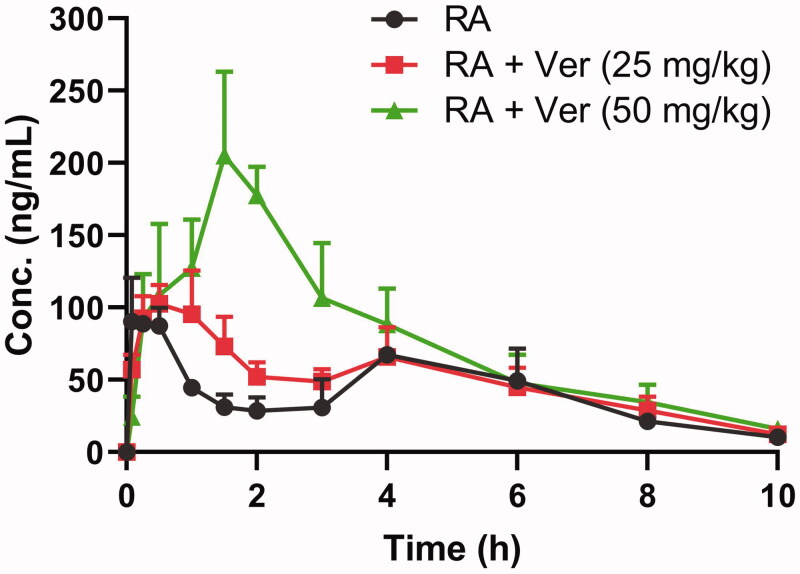
The pharmacokinetic profiles of rotundic acid (RA) in rats after the oral administration of 10 mg/kg RA with or without verapamil (Ver) pre-treatment (25 and 50 mg/kg). Each point represents the mean ± SD of six determinations.

The mean plasma concentration-time curves of RA in the control group (equal amounts of vehicle) and pre-treatment groups (pre-treatment of verapamil at 25 or 50 mg/kg) are shown in Figure 1. The differences among the control group and pre-treatment with verapamil groups were significantly different when *p*-values were less than 0.05. The pharmacokinetic parameters are presented in Table 1.

**Table 1.** The comparison of main pharmacokinetic parameters of rotundic acid in rats after a single oral administration at dose of 10 mg/kg with or without pre-treatment of verapamil at 25 and 50 mg/kg (mean ± SD, *n* = 6).

Parameter	Control	Pre-treatment of verapamil (25 mg/kg)	Pre-treatment of verapamil (50 mg/kg)
*C*_max_ (ng/mL)	108 ± 10.5	117 ± 14.9	219 ± 36.8*
*T*_max_ (h)	0.292 ± 0.228	0.625 ± 0.306	1.17 ± 0.516*
AUC_0–24_ (ng × h/mL)	405 ± 68.2	496 ± 63.0	782 ± 115*
AUC_0–∞_ (ng × h/mL)	432 ± 64.2	539 ± 53.6*	836 ± 116*
*t*_1/2_ (h)	1.85 ± 0.390	2.44 ± 0.572	2.31 ± 0.404
*V*_d_/*F* (L/kg)	64.6 ± 22.6	66.7 ± 21.1	41.3 ± 12.3
CL/*F* (L/h/kg)	23.6 ± 3.50	18.7 ± 1.85*	12.2 ± 1.85*

*p < 0.05 indicates significant differences from the control.

As shown in Table 1, after oral administration, RA was rapidly absorbed and reached a maximum concentration of 108 ± 10.5 ng/mL at 0.292 h. Combined verapamil increased the *C*_max_, *T*_max_, AUC_0–24_, AUC_0–∞_, and decreased CL/F in a dose-dependent manner. Compared with the control group, coadministered verapamil at 25 and 50 mg/kg caused a significant increase (*p* ˂0.05) in AUC_0–∞_ from 432 ± 64.2 to 539 ± 53.6 and 836 ± 116 ng × h/mL, respectively, and a significant decrease (*p* ˂ 0.05) in CL/F from 23.6 ± 3.50 to 18.7 ± 1.85 and 12.2 ± 1.85 L/h/kg, respectively. Other pharmacokinetic parameters, C_max_, T_max_, and AUC_0–24_, showed only significant differences (*p* ˂ 0.05) at high-dose verapamil. Although the *t*_1/2_ of RA increased from 1.85 ± 0.390 to 2.44 ± 0.572 and 2.31 ± 0.404 h, respectively, the difference was not significant (*p* > 0.05) when RA was coadministered with 25 and 50 mg/kg verapamil. These results indicated the metabolism of RA was inhibited when the rats were pre-treated with verapamil, and the systemic exposure of RA was significantly increased.

### Effects of verapamil on the transport of rotundic acid across MDCKII-MDR1 and Caco-2 cells

The transport of RA with or without pre-treatment of verapamil was studied via the MDCKII-Mock, MDCKII-MDR1, and Caco-2 cell monolayer model. MTS cell-proliferation assays were performed to investigate the effects of 25 µM RA on MDCKII-Mock, MDCKII-MDR1, and Caco-2 cells. The results showed that RA did not influence the cell proliferation of the above cells. The TEER values were 196 ± 36.7, 212 ± 40.6 and 674 ± 69.5 Ω·cm^2^, and the *P*_app(AP→BL)_ of Lucifer yellow were 0.463 ± 0.0568 × 10^−6^, 0.432 ± 0.0468 × 10^−6^, and 0.268 ± 0.0346 × 10^−6 ^cm/s in the MDCKII-Mock, MDCKII-MDR1, and Caco-2 cell monolayers, respectively. These results indicated the integrity of the three cell monolayers were well. Rhodamine-123, a typical P-gp substrate, was taken to validate the efflux activity of P-gp. Bidirectional transport assays in MDCKII-Mock, MDCKII-MDR1, and Caco-2 were performed by adding compounds to both apical (AP-to-BL) and basolateral (BL-to-AP) chambers. The transport of rhodamine-123 from the BL side to the AP side was markedly higher than the opposite way in MDCKII-MDR1 cells with a net efflux ratio of 7.24, and the net efflux ratio was reduced to 1.61 by verapamil (Table 2). The efflux ratio of Rhodamine-123 was 15.6 in the Caco-2 cells, and it was decreased to 4.86 when verapamil was present. The results indicated that the efflux activity of P-gp was qualified for the experiment in MDCKII-MDR1 and Caco-2 cells.

**Table 2.** Permeability (*P*_app_), efflux ratio (*R*_E_), and net efflux ratio (N*R*_E_) of Rhodamine 123 and RA with or without P-gp inhibitor verapamil (Ver) across MDCKII-Mock and MDCKII-MDR1 cell monolayers at the incubation time of 20 min (mean ± SD, *n* = 3).

P-gp substrate	MDCKII-Mock			MDCKII-MDR1			N*R*_E_
	*P*_app (AP–BL)_^a^	*P*_app (BL–AP)_	*R*_E_	*P*_app (AP–BL)_	*P*_app (BL–AP)_	*R*_E_	
Rhodamine 123	0.378 ± 0.0314	0.481 ± 0.0998	1.27	0.193 ± 0.0535	1.78 ± 0.293	9.20	7.24
Rhodamine 123 + Ver	0.409 ± 0.0686	0.538 ± 0.0642	1.32	0.315 ± 0.0320	0.673 ± 0.150	2.13	1.61
RA	6.07 ± 0.886	6.72 ± 2.03	1.11	4.22 ± 0.611	12.1 ± 1.55	2.87	2.58
RA + Ver	6.52 ± 0.864	6.23 ± 0.895	0.955	7.18 ± 0.972	9.80 ± 1.63	1.36	1.42

^a^ *P*_app_: × 10^−6 ^cm/s.

**Figure 2.**
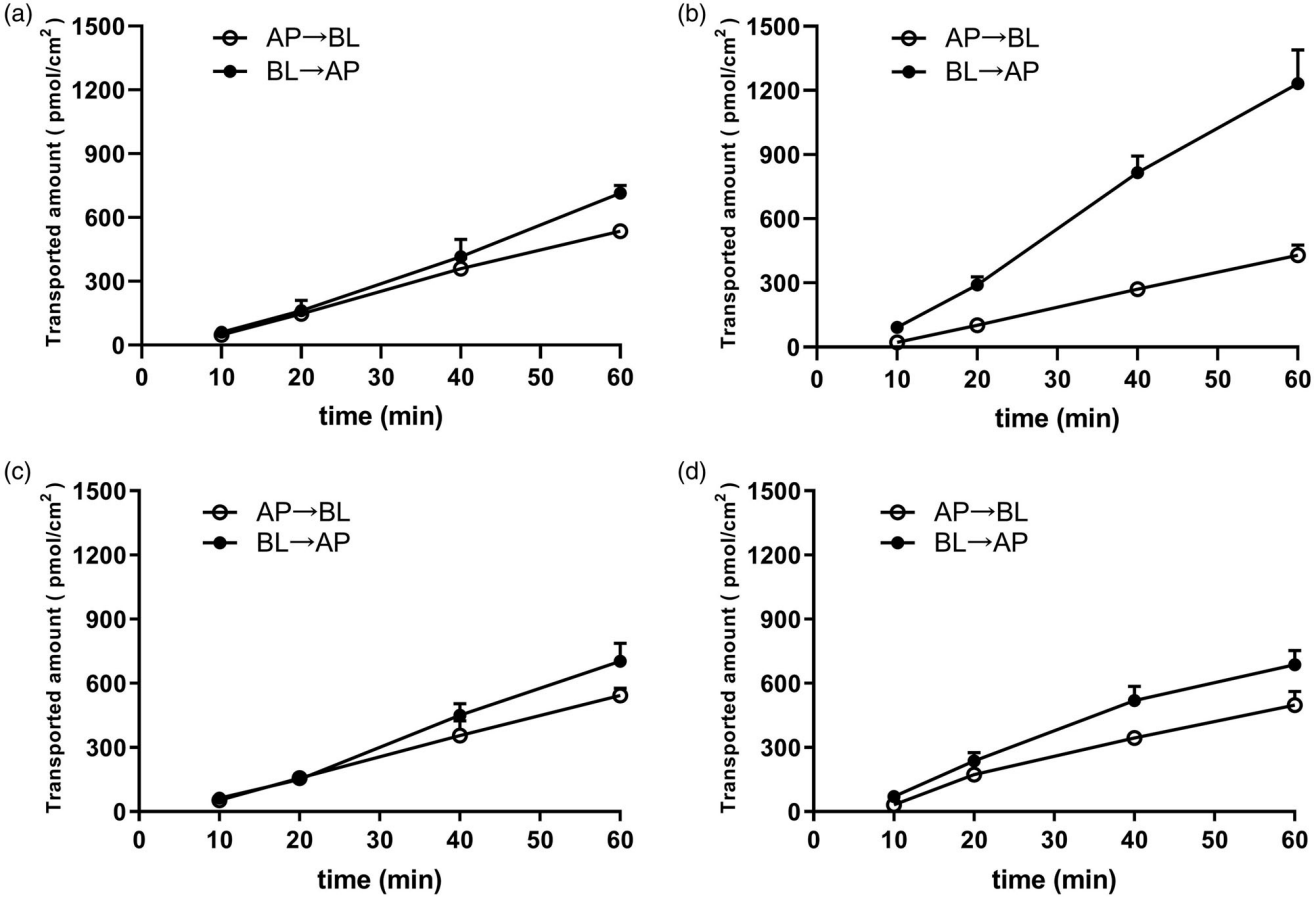
Bidirectionally transported amounts (pmol/cm^2^) of RA across (a, c) MDCKII-Mock and (b, d) MDCKII-MDR1 monolayers from 10 to 60 min. (a, b), 25 μM RA. (c, d) 25 μM RA + 50 μM Ver. Each point represents the mean ± SD of three determinations. AP: apical side; BL: basolateral side.

**Figure 3.**
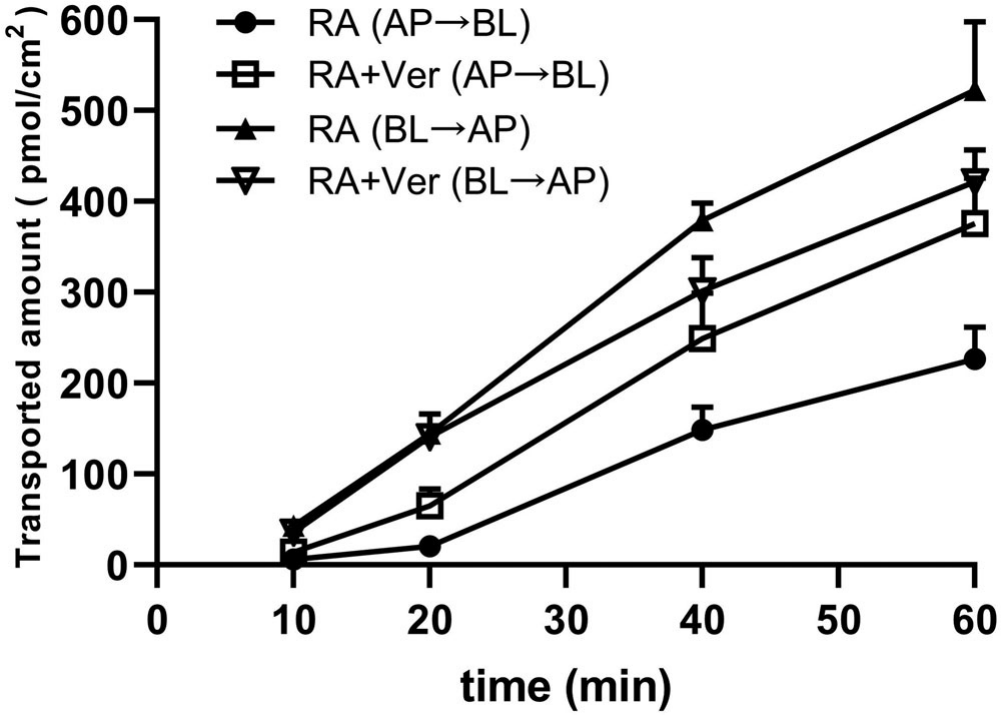
Effects of verapamil on the transported amount of RA across Caco-2 cell monolayer in different time points with a concentration of 25 μM. Each point represents the mean ± SD of three determinations. AP: apical side; BL: basolateral side.

P-gp substrate assessment for RA was studied on MDCKII-Mock and MDCKII-MDR1 cells. As shown in Figure 2, the amount of RA bidirectionally transported was measured. The vectorial transport of RA was observed in the MDCK-MDR1 cell monolayer with a net efflux ratio of 2.58 compared with the MDCKII–mock cell (Figure 2(a,b); Table 2). With the pre-treatment of verapamil (50 µM), the efflux transport of RA was significantly inhibited, and the net efflux ratio of RA were reduced to 1.42 (Figure 2(c,d), Table 2). These results suggested that P-gp mediated the efflux transport of RA. To investigate the effect of verapamil on intestinal transport of RA, both *P*_app (AP→BL)_ and *P*_app (BL→AP)_ were measured using the Caco-2 cell model. The results were shown in Table 3 and Figure 3. The transported amounts of 25 µM RA increased with time (Figure 3), and the *P*_app (BL→AP)_ values of RA was 2-fold greater than its *P*_app (AP→BL)_ values at 10–60 min. In the presence of 50 µM verapamil, the *P*_app_ values from the AP side to the BL side significantly increased (*p* < 0.05), whereas those obtained from the BL side to the AP side decreased slightly, and was not significant except for 40 min. The efflux ratio of RA decreased significantly with 50 µM verapamil pre-treatment. In short, the absorption of RA was increased with the pre-treatment of verapamil by inhibiting P-gp mediated intestinal efflux of RA.

**Table 3.** Time course of rotundic acid transport across the Caco-2 cell monolayer with or without P-gp inhibitor verapamil (mean ± SD, *n* = 3).

Time (min)	*P*_app_ (× 10^−6^ cm/s), RA			*P*_app_ (× 10^−6^ cm/s), RA + Ver		
	AP→BL	BL→AP	*R*_E_	AP→BL	BL→AP	*R*_E_
10	0.468 ± 0.0625	3.25 ± 0.350	6.93	1.07 ± 0.212*	2.73 ± 0.348	2.55
20	0.776 ± 0.128	5.45 ± 0.806	7.02	2.45 ± 0.707*	5.32 ± 0.306	2.17
40	2.80 ± 0.467	7.13 ± 0.360	2.55	4.69 ± 0.945*	5.68 ± 0.683*	1.21
60	2.84 ± 0.441	6.56 ± 0.941	2.31	4.71 ± 0.524*	5.29 ± 0.438	1.12

*p < 0.05, compared with the RA group.

### The metabolism of RA through rhCYPs

**Figure 4.**
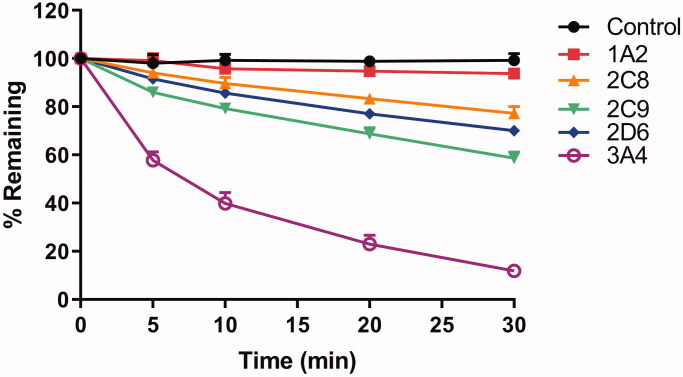
Time dependent metabolic depletion of RA against five rhCYPs. Initial concentration of RA was 2 µM, and protein concentration of rhCYPs was 25 pM. Each point represents the mean ± SD of three determinations.

To identify CYP enzymes involved in the metabolism of RA, the compound was incubated with five rhCYPs (1A2, 2C8, 2C9, 2D6 and 3A4) at a concentration of 2 µM and different time periods. The results were shown in Figure 4. No obvious degradation of RA was found in the negative control using heat-inactivated rhCYP3A4, showing that RA was stable in the incubation system. Based on the substrate (RA) depletion method, after 30 min exposure to five rhCYPs, RA was hardly metabolized by CYP1A2, only a few metabolites were produced in CYP2C8 and 2D6 (<30% RA converted), and moderate metabolite in CYP2C9 (41.4% RA converted). In contrast, RA was principally metabolized by CYP3A4 (88.2% RA converted). These data suggest that RA is a substrate for CYP2C8, 2C9, 2D6, and 3A4. Among these, CYP3A4 was found to be predominant in the metabolism of RA.

### Effects of verapamil on the metabolic stability of rotundic acid in rat liver microsomes

**Figure 5.**
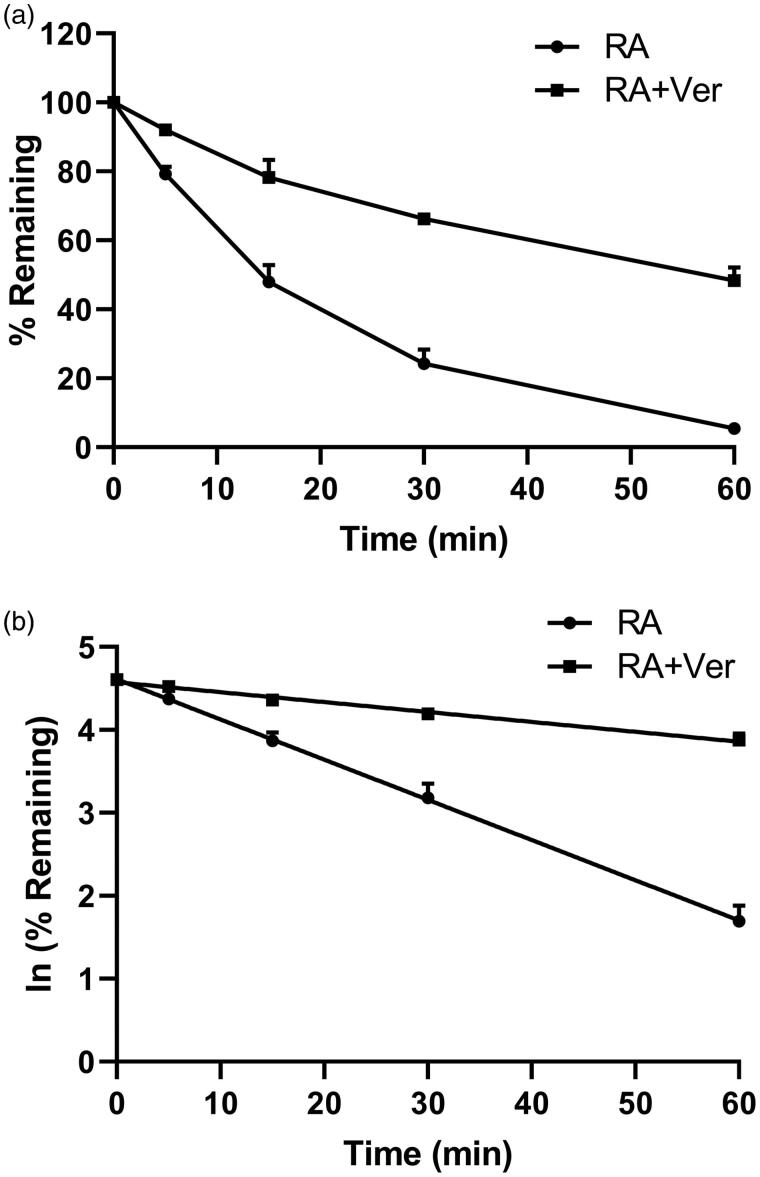
*In vitro* metabolic stability of rotundic acid (RA) in rat liver microsomes with or without treatment of verapamil (Ver). (a) Plots of relative remaining substrate level versus time. (b) The natural logarithm (ln) of relative remaining substrate level versus time. Each point represents the mean ± SD of three determinations.

The effects of verapamil on the metabolic stability of RA were investigated using rat liver microsomes incubation systems. No obvious degradation of RA was found in the negative control using heat-inactivated microsomes, showing that RA was stable in the incubation system. A comparison of RA elimination rate in RLMs with or without treatment of verapamil is presented in Table 4 and Figure 5. These results indicated that RA degraded quickly in RLMs, the *t*_1/2_ of RA was 14.3 ± 0.941 min, while it was prolonged (58.2 ± 5.17 min) in the presence of verapamil, and the difference was significant (*p* < 0.05). These results combined with the metabolic stability of RA in rhCYPs indicated that verapamil could slow down the metabolism of RA in RLMs mainly via the inhibition of CYP3A activity. Moreover, the CL_int_ of RA was reduced from 48.5 ± 3.18 to 12.0 ± 1.06 µL/min/mg protein by verapamil. The prolonged *in vitro* half-life and the reduced intrinsic clearance verified the results of the pharmacokinetic study. It suggested that the metabolic stability of RA in RLMs was enhanced by verapamil.

**Table 4.** The comparison of *in vitro* metabolic parameters of rotundic acid in rat liver microsomes with or without pre-treatment of verapamil (mean ± SD, *n* = 3).

Parameter	Control	Pre-treatment of verapamil
*k*_e_ (min^−1^)	0.0485 ± 0.00318	0.0120 ± 0.00106*
*t*_1/2_ (min)	14.3 ± 0.941	58.2 ± 5.17*
CL_int_ (µL/min/mg protein)	48.5 ± 3.18	12.0 ± 1.06*

*p < 0.05 indicates significant differences from the control.

## Discussion

Rotundic acid, a plant-derived pentacyclic triterpene, is widely present in medicinal and edible plants (Saimaru et al. 2007; Yang, Li, Ruan, Tong, et al. 2018). RA has been reported to possess anti-inflammatory and cardio-protective abilities. However, due to its poor bioavailability just like PTAs, the further development and potential therapeutic application of RA was limited. To our best knowledge, the reasons for the low bioavailability of PTAs are mainly due to the P-gp mediated drug effluxes and/or CYP3A4 mediated metabolisms (Zhao et al. 2012; Yang et al. 2017). We speculated that the poor bioavailability of RA is related to the same reason. Verapamil, commonly used to treat high blood pressure, could improve their absorption of the drugs with poor bioavailability, via inhibiting the activity of CYP3A4 and P-gp (Zhu et al. 2017; Zhou et al. 2019; Xing et al. 2020). Whether verapamil has the same effect on RA is still unclear. Therefore, the effect of verapamil on the pharmacokinetics of RA in rats was investigated and the main mechanism was clarified. Furthermore, we investigated the transporter-enzyme interaction of RA *in vivo* that could contribute to its low oral bioavailability.

In the present study, the pharmacokinetic experiments showed RA was quickly absorbed and reached the peak plasma concentration at approximately 15 min after oral administration, and rapidly eliminated with the *t*_1/2_ of approximately 2 h. The plasma exposure of RA was increased by the administration of verapamil, as the value of *C*_max_, AUC_0–24_, AUC_0–∞_, *T*_max_ increased, and the CL/F of RA decreased, which indicated verapamil could inhibit the metabolism of RA. Transporters and metabolic enzymes that participate in the absorption and metabolism of many clinical drugs in the intestine and liver might influence the pharmacokinetics of co-administrated drugs and result in adverse effects (Shi et al. 2016; Zhao et al. 2019). To investigate the potential mechanism of the effect of verapamil on RA and the reason for its poor bioavailability, the MDCKII-Mock, MDCKII-MDR1, and Caco-2 cell monolayer models, rhCYPs (1A2, 2C8, 2C9, 2D6 and 3A4), and rat liver microsomes were used. The results show that RA is a substrate for CYP2C8, 2C9, 2D6, and 3A4. Especially, it was demonstrated that RA was mainly metabolized by CYP3A4 in our studies. Through the MDCKII-Mock and MDCKII-MDR1 cell line assay, RA was found to be transported by P-gp.

From the transport results of RA in Caco-2 cell monolayer model, it was inferred that the efflux transporter might be involved in the transport of RA, as the efflux of RA was much higher than the influx. The uptake of RA (25 µM), when it was concurrently administered with verapamil (50 µM), was greater than that in the RA-only treatment. These results were consistent with the animal experiment results and revealed that the uptake interactions of RA and verapamil also existed in Caco-2 cell when co-administered. The efflux protein transporters exert critical roles in the absorption of natural compounds (Mahringer et al. 2010; Yan et al. 2017; He et al. 2020). In this study, verapamil decreased the efflux ratio of RA, and the absorption of RA was enhanced. It could be concluded that verapamil may inhibit the intestinal efflux of RA. The RLMs were employed to investigate the effect of verapamil on the metabolic stability of RA. In RLMs, the *t*_1/2_ was prolonged and CL_int_ of RA were decreased by the pre-treatment with verapamil. Therefore, we inferred that verapamil might affect the pharmacokinetics of RA by inhibiting the hepatic metabolism of RA.

In summary, the *in vitro* tests for permeability across MDCKII-MDR1 cell monolayers and metabolic stability in rhCYPs showed that RA was a substrate of both P-gp and CYP3A4. Additionally, verapamil could inhibit the efflux of RA in the intestine and metabolism of RA in the liver, as the *R*_E_ and CL_int_ of RA decreased significantly. These results were also confirmed *in vivo* pharmacokinetic study in which verapamil could increase the systemic exposure of RA in rats when they are co-administrated. It could be inferred that CYP3A mediated metabolism and the participation of the efflux transporter P-gp are the main reasons for the low bioavailability of RA. In addition, verapamil might exert these effects mainly through increasing the absorption of RA by inhibiting the activity of P-gp, and/or by slowing down the metabolism of RA in rat liver by inhibiting the activity of CYP3A. However, the poor solubility and dissolution rate may also influence the bioavailability and pharmacodynamics of RA (Peterson et al. 2019). Therefore, further studies should give more attention to the solubility and dissolution of drugs.

## Conclusions

This study suggests that the reason for the poor bioavailability of RA due to both CYP3A mediated metabolism and P-gp mediated drug effluxes, and co-administration of verapamil with rotundic acid had a significant effect on oral pharmacokinetics of RA, which increased the systemic exposure of RA. These results of this experiment indicated that verapamil could increase the absorption of RA via inhibiting the activity of P-gp in Caco-2 cell model and slow down the metabolism of RA in rat liver by inhibiting the activity of CYP3A. Therefore, the combination of these two drugs should be given special attention in the clinic. However, the roles of other metabolic enzymes or other transporters might be affected by the bioavailability and pharmacodynamic effect of RA, which requires further studies to verify.
